# Effect of Copper and Titanium-Exchanged Montmorillonite Nanostructures on the Packaging Performance of Chitosan/Poly-Vinyl-Alcohol-Based Active Packaging Nanocomposite Films

**DOI:** 10.3390/foods10123038

**Published:** 2021-12-07

**Authors:** Constantinos E. Salmas, Aris E. Giannakas, Maria Baikousi, Eleni Kollia, Vasiliki Tsigkou, Charalampos Proestos

**Affiliations:** 1 Department of Material Science and Engineering, University of Ioannina, 45110 Ioannina, Greece; mariabaikousi@gmail.com; 2 Department of Food Science and Technology, University of Patras, 30100 Agrinio, Greece; agiannakas@upatras.gr; 3 Laboratory of Food Chemistry, Department of Chemistry, National and Kapodistrian University of Athens Zografou, 15771 Athens, Greece; elenikollia@chem.uoa.gr (E.K.); vtsigkou@chem.uoa.gr (V.T.)

**Keywords:** copper, titanium, montmorillonite, chitosan, poly-vinyl-alcohol, nanocomposites, active packaging

## Abstract

In this study, CuMt and TiMt montmorillonites were produced via an ion-exchange process with Cu^+^ and Ti^4+^ ions. These nanostructured materials were characterized with X-ray diffraction (XRD) and fourier transform infrared spectroscopy (FTIR) measurements and added as nanoreinforcements and active agents in chitosan (CS)/poly-vinyl-alcohol (PVOH)-based packaging films. The developed films were characterized by XRD and FTIR measurements. The antimicrobial, tensile, and oxygen/water-barrier measurements for the evaluation of the packaging performance were carried out to the obtained CS/PVOH/CuMt and CS/PVOH/TiMt films. The results of this study indicated that CS/PVOH/CuMt film is a stronger intercalated nanocomposite structure compared to the CS/PVOH/TiMt film. This fact reflected higher tensile strength and water/oxygen-barrier properties. The antibacterial activity of these films was tested against four food pathogenic bacteria: *Escherichia coli*, *Staphylococcus aureus*, *Salmonella enterica* and *Listeria monocytogenes*. Results showed that in most cases, the antibacterial activity was generated by the CuMt and TiMt nanostructures. Thus, both CS/PVOH/CuMt and CS/PVOH/TiMt films are nanocomposite candidates with very good perspectives for future applications on food edible active packaging.

## 1. Introduction

Presently, following the cyclic economy and sustainability spirit, food packaging changes from passive packaging which acts as a simple container and/or insulating barrier from the outside environment to active packaging, which absorbs substances that interact with food. Active packaging systems are used with foods, pharmaceuticals, and other products to extend their shelf-life, monitor their freshness and quality, and increase their safety and accessibility by consumers. Such systems possess active functions further than the commonly used inert, passive protection atmosphere. Active or intelligent packaging systems measure specific properties of a product to sense parameters critical for its condition. This information can be promoted to the consumer and/or ignite specific packaging safety functions [1]. This leads to an increase in the shelf-life and to extra protection of the quality of products [2,3]. The recent application by researchers of nanotechnology techniques on active packaging material development have shown great potential to enhance the packaging’s properties and to add to it new functionalities such as active features. Moreover, the environmental issues that arise from the use of plastic packaging materials increase the effort to apply nanotechnology techniques for the development of alternative sustainable, bio-based packaging materials. One of the most promising bio-based materials for active packaging applications is chitosan (CS) [4,5]. CS is a derivative from chitin which can be produced via a deacetylation process of chitin. Chitin is the second more abundant biopolymer.

The innovative concept to develop antimicrobial-active films for food packaging applications originated from consumers’ expectations for microbiologically safer food [6]. Several studies were carried out using different antimicrobial agents for this purpose [6]. It is well known that metallic ions, such as silver [7,8,9], copper [10], and zinc [11], titanium [12,13], and iron [14] exhibit strong inhibitory and bactericidal properties for a wide variety of bacteria. Metallic ions which are stabilized in supported nanomaterials such as nanoclays can be incorporated directly in either polymer or biopolymer matrices. This technique succeeds to control the release of the exchanged ions from the synthetic clay and thus to achieve long-term antibacterial effectiveness. Although copper-loaded mineral substrates have not been investigated enough as active food packaging materials, presently silver-loaded clays dominate other antibacterial chemical ions as antibacterial agents [15]. The immobilization procedure of Cu ions into Mt interlayer space as well as their antimicrobial properties were extensively reported [16,17,18]. In contrast to other antimicrobial metals, copper exhibits a wide variety of actions against bacteria and molds, and because of this, it attracts big research interest. On the other hand, titanium-exchanged montmorillonite (TiMt) was synthesized and investigated mainly as a catalyst [19,20,21]. No reports were found concerning the investigation of TiMt as an active antimicrobial agent. Some recent papers report the addition of copper-exchanged montmorillonite (CuMt) in cellulose acetate [22], gelatin [23], and low-density polyethylene [24] to produce antimicrobial-active packaging films. Bruna et al. [24] developed novel CuMt/Low-density polyethylene packaging films and found that the antibacterial reduction of *Escherichia coli* increased by increasing CuMt content. More recently, Bruna et al. [22] developed CuMt/cellulose antimicrobial films and reported a 98% reduction of *Escherichia coli.* Martucci et al. [23] prepared CuMt and embed it into a bovine gelatin matrix via a dissolution-intercalation method. The final product of this process was a novel antibacterial nanocomposite film with a stronger effect on *L. monocytogenes* than on *E. coli* pathogen. Nevertheless, major issues on active packaging are not solved yet and a lot of research effort still exists globally to develop novel active packaging materials using low cost, biodegradable, and environmentally friendly precursors. The criteria for the success of such scientific attempts are the extension of shelf-life of products, the antimicrobial and antioxidant activity of the packaging, the enhanced mechanical properties, the increased oxygen and water-vapor barrier of the packaging. This is the aim of our work here.

## 2. Materials and Methods

### 2.1. Materials

Sodium exchanged montmorillonite (NaMt) was supplied by Sigma-Aldrich and produced by Nanocor Inc. (2870 Forbs Avenue, Hoffman Estates, Chicago, IL, USA). The code name of this material was Nanomer^®^ PGV and its mass density was 2.6 g/cm^3^. The CEC value was 145 meq/100 g, and its chemical composition was 19.6% Al_2_O_3_, 62.9% SiO_2_, 3.05% MgO, 3.35% Fe_2_O_3_, 1.68% CaO, and 1.53% Na_2_O. Poly(vinyl alcohol) (PVOH) was purchased by Sigma-Aldrich. This chemical exhibit a low molecular weight (13.000−23.000). Furthermore, its hydrolysis degree was around 87−89%. Finally, reagents such as 37% ACS hydrochloric acid (7647-01-0), silver nitrate (7761-88-8), and titanium tetrachloride (7550-45-0) were also supplied by Sigma-Aldrich.

#### 2.1.1. Preparation of CuMt and TiMt Ion-Exchanged Nanoclays

Cu^+^ exchanged montmorillonite nanoclay was prepared as follows: 5 g of commercial NaMt were dispersed in 200 mL of 0.05 mol/L CuSO_4_ solution. Using 0.05 M HCl solution, the pH value was adjusted to 5.0. The dispersion was kept for 24 h under constant stirring (500 rpm) at room temperature in a hot plate and then centrifuged at 5000 rpm for 5 min. The recovered sediment was washed with distilled water until no acid in the supernatant was detected, dried at 80 °C overnight in an air-circulating oven, and finally grounded in fine powder.

The Ti^4+^-exchanged montmorillonite (TiMt) nanoclay was produced by reacting NaMt with 1 M TiCl_4_ solution. An amount of 5 g of commercial NaMt was dispersed in 200 mL of distilled water. The NaMt suspension was placed in an ice bath to keep the temperature constant and under 4 °C. An amount of TiCl_4_ solution equal to five times the amount of the CEC (145 meq/100 g) was used to treat the clay suspension [25]. The recovered TiMt sediment was washed with distilled water until tests with AgNO_3_ indicate that no chloride remains.

#### 2.1.2. Preparation of CS/PVOH/CuMt and CS/PVOH/TiMt Active Nanocomposite Films

All films were prepared via a reflux-heat pressing method according to our previous publications [26,27,28,29]. The compounding of CS/PVOH blends and CS/PVOH/clay nanocomposites films was carried out using a 2 *w*/*v*% CS solution which was prepared by dissolving the CS powder in 1 *v*/*v*% glacial acetic acid aqueous solution (HAc), under vigorous stirring for 24 h at 70 °C. Then the solution (pH 4.4) was cooled down regularly at room temperature. An appropriate amount of the 2 *w*/*v*% CS solution was mixed with hot water containing PVOH to achieve final blends with 20 wt.% PVOH content. The mixtures were refluxed under stirring for 2 h. Obtained solutions were cast onto plastic dishes (12 cm diameter). The castings were dried at ambient conditions (22 °C) for 5 days and then the produced films were peeled off. After drying, films were pressed for 2 min at 120 °C under 3 MPa constant pressure, using a hydraulic press with heated platens. This process reduces the films’ moisture content and improves their mechanical properties because of the better packing of the CS chains in the film [27,30]. For the preparation of the CS/PVOH/clay nanocomposites, appropriate amounts of CuMt and TiMt nanostructures were dispersed in 20 mL distilled water, and the obtained suspensions were added in the PVOH solutions to achieve final 3, 6 wt.% nanostructure contents. The obtained PVOH/NaMt, PVOH/CuMt, and PVOH/TiMt suspensions were left under vigorous stirring for 2 h, mixed with 2 *w*/*v*% CS solution, and refluxed under stirring for 4 h. Obtained dispersions were dried and pressed as described above. Symbol of samples and exact quantities used for the preparation of the films are presented in Table 1.

### 2.2. XRD Analysis

CS/PVOH/CuMt and CS/PVOH/TiMt active films, as well as CuMt and TiMt ion-exchanged nanostructured powders, were characterized using an XRD instrument. A Brüker D8 Advance X-ray diffractometer (Brüker, Analytical Instruments, S.A., Athens, Greece) which was equipped with a LINXEYE XE High-Resolution Energy-Dispersive detector was used to produce the XRD spectra. The measurements of the CuMt and TiMt ion-exchanged nanostructure powders were carried out in the range 2θ = 2°–50° while the measurements of the CS/PVOH/CuMt and CS/PVOH/TiMt active films were carried out in the range 2θ = 2°–40°. The increment for both groups of samples was 0.03.

### 2.3. FTIR Spectrometry

The measured infrared (FTIR) spectra were obtained following a scanning frequency in the range of 4000–400 cm^−1^, while 32 scans at 2 cm^−1^ resolution were used for the final average value of each measurement. An FT/IR-6000 JASCO Fourier transform spectrometer (JASCO, Interlab, S.A., Athens, Greece) was used to investigate the chemical structure of both CuMt and TiMt ion-exchanged nanostructures.

### 2.4. Tensile Properties

The ASTM D638 method was followed to measure the tensile properties of CS/PVOH/CuMt and CS/PVOH/TiMt active films. A Simantzü AX-G 5kNt instrument (Simandzu. Asteriadis, S.A., Athens, Greece) was used to tension three to five samples of each film at an across head speed of 2 mm∙min^−1^. The sample shape was a dumbbell with gauge dimensions of 10 × 3 × 0.22 mm. The stress, strain, and modulus of elasticity values were calculated using the recorded values of the force (N), the deformation (mm), and the gauge dimensions.

### 2.5. Water Sorption

The dry mass of film samples (20 × 20 mm) was calculated by weighting after 24 h desiccation under vacuum. Such dry films were stored at T = 25 °C for 3 h in closed beakers containing 50 mL of deionized water. The saturation stage with water of these samples was determined by sequential periodical weighting and the total water sorption was calculated using Equation (1):

(1) $$W.G.\left(\%\right)=\frac{m_{wet}-m_{dry}}{m_{dry}}\times 100$$

where $m_{wet}$ is the weight of the saturated sample, $m_{dry}$ is the weight of the dry sample, and *W.G.* is the Water Gain.

### 2.6. Water-Vapor Diffusivity

Water-Vapor Transmission Rate (WVTR) was estimated according to the ASTM E96/E 96M-05 method. A handmade apparatus was used for such measurements of CS/PVOH/CuMt and CS/PVOH/TiMt films. Experiments were carried out at 38 °C and 50% RH. The methodology was described extensively in the literature [31,32,33,34,35,36,37]. The 2.5 cm diameter and 0.09 mm average thickness film was placed on the top of a one-open end cylindrical tube made of plexiglass which contained dried silica gel inside and was sealed by a rubber O-ring. The test tube was placed in a glass desiccator which contained 200 mL saturated magnesium nitrate solution (50% relative humidity (RH) at 38 °C). Test tubes were weighed periodically for 24 h and the *WVTR* [g/(cm^2^∙s)] was calculated according to the following equation:

(2) $$WVTR=\frac{\Delta G}{t\cdot A}$$

where: Δ*G* (g) is the increase of weight of the tested tubes, *t* (s) is the time pass, Δ*G*/*t* (g/s) is the water transmission rate through the film which is calculated by the slope of the linear function Δ*G* = f(*t*), and *A* (cm^2^) is the permeation area of the film. Additionally, the weight of the tested films was measured before and after the WVTR test to exclude any absorption phenomena of humidity by the film.

For diffusion process through a membrane, Fick law [38] calculates the specific mass flow rate via the following equation:

(3) $$\frac{J}{A}=D\frac{\Delta C}{\Delta x}$$

where *J* (g/s) is the mass flow rate of a component through the membrane, *A* (cm^2^) is the membrane cross-sectional area permeated by this component, Δ*C* (g/cm^3^) is the concentration gradient of this component in the two sides of the membrane, and Δ*x* (cm) is the membrane thickness.

Assuming that in our apparatus silica gel on one side absorbs the permeated water vapor totally and given that according to the ASTM E96/E 96M-05 method the humidity concentration in the opposite side of the film is 22.86747 g/cm^3^ (50% RH at 38 °C), then Δ*C* = 22.86747 g/cm^3^. For *WVTR* = *J*/*A* and combine Equations (2) and (3) we can calculate the diffusion coefficient *D* (cm^2^/s) for every film as follows:

(4) $$D_{WV}=WVTR\cdot \frac{\Delta x}{\Delta C}$$

where *WVTR* [g/(cm^2^∙s)] is the water-vapor transmission rate, Δ*x* (cm) is the film thickness, and Δ*C* (g/cm^3^) is the humidity concentration gradient in the two opposite sides of the film.

### 2.7. Oxygen Permeability

The oxygen transmission rate (OTR) was measured using an oxygen permeation analyzer (8001, Systech Illinois Instruments Co., Johnsburg, IL, USA). The examined samples were tested at 23 °C and 0% RH according to the ASTM D 3985 method. OTR values were measured in cc O_2_/m^2^/day.

According to the literature [39], gas permeability through polymers could be expressed as follows:

(5) $$\frac{J}{A}=Pe_{gas}\cdot \frac{\Delta C}{\Delta x}$$

where *J*/*A* [mol/(cm^2^∙s)] is the specific amount of gas pass through the membrane, *Pe_gas_* (cm^2^/s) is the permeability coefficient, Δ*C* (mol/cm^3^ STP) is the pressure gradient in the two opposite sides of the membrane, and Δ*x* (cm) is the membrane thickness.

Rearrange Equation (5) we can express the permeability coefficient as follows:

(6) $$Pe_{gas}=\frac{J}{A\cdot \Delta C}\cdot \Delta x$$

By carrying out a dimensional analysis, the term *J*/(A·Δ*C*) [cm^3^ STP/(cm^2^∙s)] is equal to the OTR measurements. Thus, in our case for oxygen gas,

(7) $$Pe_{O2}=OTR\cdot \Delta x$$

The oxygen permeability coefficient values (*Pe_O2_*) of the tested samples were calculated by multiplying the *OTR* values with the average film thickness of 0.035 mm. The *OTR* value for each kind of film resulted from the mean value of measurements of three pieces.

### 2.8. Antimicrobial Activity Tests

Antimicrobial activity tests of films were carried out using the agar diffusion method [40]. The films were tested against Gram-negative bacteria *Escherichia coli* (ATCC 25922)*, Salmonella enterica* subsp. *enterica* (DSMZ 17420), and Gram-positive bacteria *Staphylococcus aureus* (DSMZ 12463)*, Listeria monocytogenes* (DSMZ 27575). The bacteria isolates were obtained from the Institute of Technology of Agricultural Products, ELGO-DEMETER, Lykovryssi, Greece. Bacteria colonies were diluted in Mueller-Hinton broth and cultured overnight to achieve a range of 10^7^–10^8^ CFU mL^−1^. The 24 h old cultures of bacteria were swabbed on Mueller-Hinton agar plates by rotating the plate every 60° to ensure homogeneous growth. Films were cut into a disc form of 6 mm diameter using a circular knife and sterilized by a UV lamp. Discs were placed on Mueller-Hinton inoculated dishes and incubated at 37 °C for 24 h. The diameter of inhibitory zones, as well as the contact area of the films with agar surface, was measured. The experiment was performed in triplicates.

## 3. Results

### 3.1. XRD

**Figure 1:**
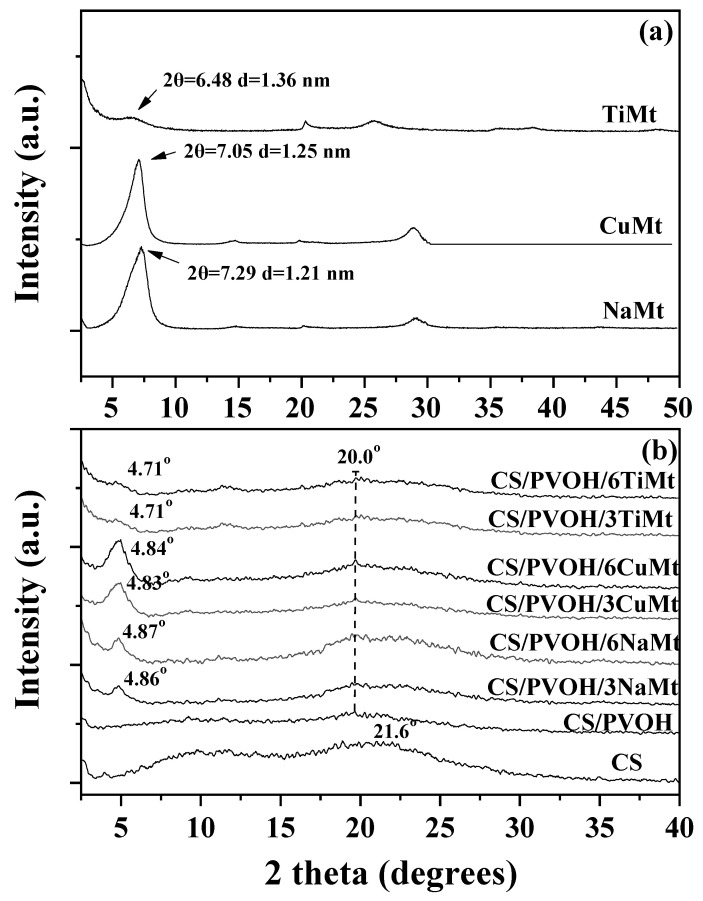
X-ray Diffraction (XRD) plots of (**a**) NaMt, CuMt, and TiMt nanoclays and (**b**) pure CS, CS/PVOH films and CS/PVOH/NaMt, CS/PVOH/CuMt, and CS/PVOH/TiMt nanocomposite films. NaMt is sodium-montmorillonite composite; CuMt is copper-montmorillonite composite; TiMt is titanium-montmorillonite composite; CS is chitosan; PVOH is Poly-vinyl-alcochol; CS/PVOH/NaMt is the final film containing chitosan/sodium-montmorillonite nanostructure in poly-vinyl-alcochol matrix; CS/PVOH/CuMt is the final film containing chitosan/copper-montmorillonite nanostructure in poly-vinyl-alcochol matrix; CS/PVOH/TiMt is the final film containing chitosan/titanium-montmorillonite nanostructure in poly-vinyl-alcochol matrix.

X-ray diffraction patterns of raw NaMt and modified CuMt, TiMt nanoclays were used to determine the variations in the basal d_001_-spacing due to cation switching (Figure 1a). NaMt exhibits a diffraction peak at 2θ = 7.44° which according to Bragg’s equation corresponds to the basal interlayer d_001_-spacing of 1.19 nm. After the treatment of the raw NaMt with HCl and CuSO_4_ the XRD measurements of the obtained CuMt material show that the layered structure is retained and the basal spacing of (d_001_) was estimated to be 1.25 nm. The increase of the interlayer distance in CuMt nanoclays is attributed to acid treatment [23] and/or to swelling/hydration of the nanoclay [41]. The comparison of the TiMt XRD plot with NaMt plot clearly shows that the TiMt is less crystalline than the NaMt. On the other hand, the TiMt diffraction peak is observed at 2θ = 7.44° which corresponds to a basal interlayer space of 1.26 nm according to Bragg’s equation. The results presented here are following the previous report [25]. In this work, a decrease in crystallinity of TiMt nanoclays was also observed, and the increase in basal space was attributed to the higher hydrated ionic radius of the Ti^4+^ ion as compared to Na^+^ ion [25].

Figure 1b shows the XRD patterns of pure CS film, CS/PVOH blend film, as well as XRD plots of CS/PVOH/nanoclay nanocomposite film. Pure CS sample presents two wide reflections, at around 2θ = 10° and around 2θ = 21.6°. This pattern indicates the existence of small and imperfect crystals [26,42]. The peak of neat CS at around 2θ = 10° almost disappears from the XRD pattern of the CS/PVOH blend film while CS’s peak at 2θ = 21.6° is shifted in smaller angles at around 2θ = 20°. This peak shift is indicative of chain interactions between CS and PVOH and suggests that the PVOH molecules expand the free space in the CS chain [27,43].

For all CS/PVOH/nanoclay films, XRD plots show a clear shift of the NaMt, CuMt, and TiMt basal space diffraction peak into smaller 2 theta values. For CS/PVOH/NaMt films the basal interlayer space diffraction is observed at around 4.7–4.8°. For CS/PVOH/CuMt films the basal interlayer space diffraction is observed at around 4.83–4.84°. Finally, for CS/PVOH/TiMt films the basal interlayer space diffraction is observed 4.71°. This fact indicates that in all cases insertion of CS/PVOH layers in the NaMt, CuMt, and TiMt interlayer space occurs, and an intercalated nanocomposite structure is formed. Similar results were reported elsewhere [27,44]. CS/PVOH/NaMt and CS/PVOH/CuMt films are more intercalated nanocomposite structures than CS/PVOH/TiMt films. If packaging films are intercalated nanocomposite structures, then they exhibit improved mechanical and barrier properties.

### 3.2. FTIR

**Figure 2:**
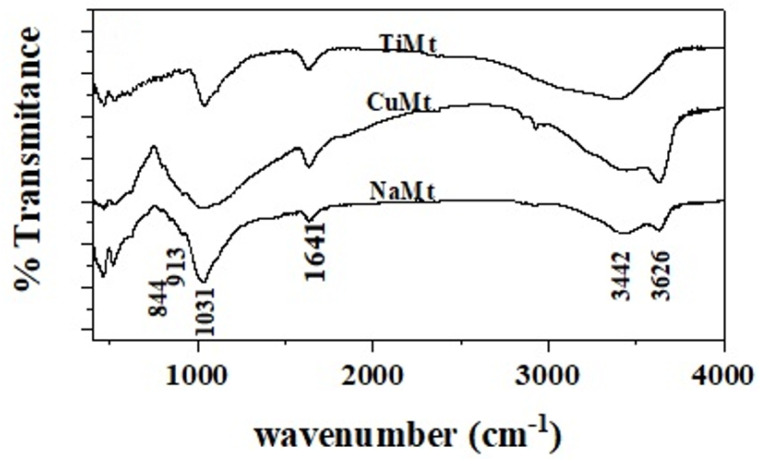
FTIR spectra of NaMt nanoclay as received and obtained CuMt and TiMt cation exchanged nanoclays. FTIR is fourier transform infrared spectroscopy.

FTIR plots of NaMt, CuMt, and TiMt nanoclays are depicted in Figure 2. FTIR plots of pure CS, CS/PVOH, CS/PVOH/NaMt, CS/PVOH/CuMt, and CS/PVOH/TiMt films are shown in Figure 1b.

The characteristic absorption band of NaMt at 3626 cm^−1^ (Figure 2) is assigned to OH group stretching, bonded with Al^3+^ cation [41]. The band at 3442 cm^−1^ is assigned to H_2_O stretching vibrations, at 1641 cm^−1^ to H_2_O bending vibrations, at 1113 cm^−1^ and 1031 cm^−1^ to SiO stretching vibrations [45]. Moreover, the three bands at 913, 879, and 844 cm^−1^ are assigned to OH bending modes. The band at 913 cm^−1^ is assigned to the bending mode of AlAl-OH, at 879 cm^−1^ is assigned to the bending mode of AlFe-OH, and finally at 844 cm^−1^ is assigned to the bending mode of FeFe-OH [41,45].

Most of the changes in the spectrum of CuMt compared to the spectrum of the NaMt were observed in the region 3700–3000 cm^−1^. As the Cu is exchanged with Na^+^, the hydration peak intensity increases. This fact is consistent with the fact that Cu^+^ holds back a higher amount of water than Na^+^ [41].

Compared TiMt FTIR spectrum with NaMt FTIR spectrum we observed that the hydration peak intensity at 3442 cm^−1^ also increases. This fact also indicates the bigger hydrated ionic radius of the Ti^4+^ ion compared to the hydrated ionic radius of the Na^+^ ion. The two above-mentioned observations were confirmed by the XRD results. Another obvious difference between TiMt and NaMt FTIR spectrum is the dramatic decrease (or almost elimination) of the reflection at 3626 cm^−1^ of TiMt which is assigned to the stretching vibration of OH groups bonded with Al^3+^. This reflection is clearly obvious in NaMt case. This almost absence of reflection at 3626 cm^−1^ in the case of TiMt nanoclays could be attributed to the higher amount of adsorbed water which maybe attenuates the stretching vibration of OH groups with Al^3+^ ions. Furthermore, a more careful observation to the FTIR spectra of such materials reveals a small shift by a few wavenumbers of the characteristic peaks of TiMt compared with NaMt. Similar FTIR spectra for TiMt and raw Mt with similar behavior reported elsewhere in the literature [46]. According to this study these shifts could be attributed to chemical changes during dehydration and pillaring metals might be present in the layers.

### 3.3. Tensile Properties

**Figure 3:**
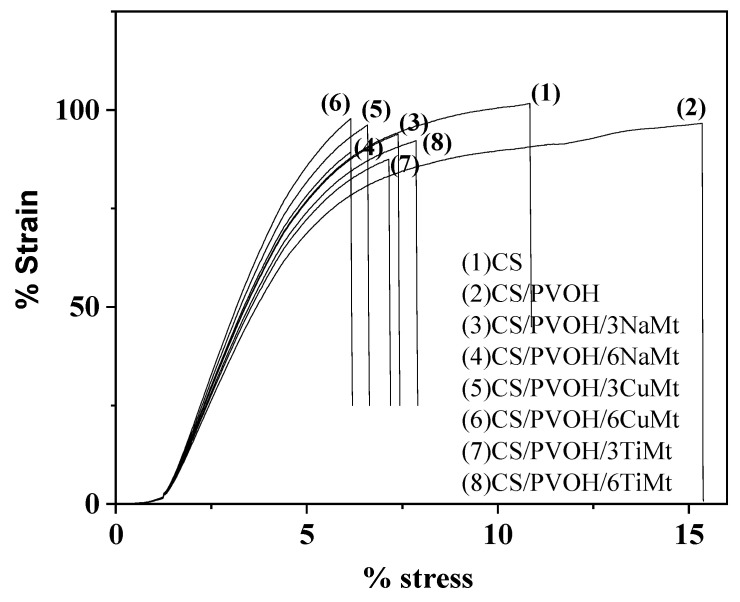
Typical stress-strain curves of all obtained films.

Typical stress-strain curves for CS, CS/PVOH, and all the obtained nanocomposite films are presented in Figure 3. The average values of the E Modulus, tensile strength and strain at break are provided in Table 2. It is obvious from Figure 3 that most of the tested films exhibit a typical semi-crystalline polymer stress-strain behavior. The addition of PVOH leads to a distinct plastic flow region with higher strain at break values and a significant decrease in stiffness and strength. These observations are following previous reports [27,47,48].

With the addition of nanoclays, a pronounced enhancement of the stiffness and strength can be observed. In most cases of the nanocomposite films, these observations are followed by a significant decrease of the strain at break values. The increase of stiffness and strength is higher in the case of NaMt and CuMt nanoclays while a small increment is observed for TiMt-based nanocomposite films. It is obvious from Table 2 that the higher the nanoclay content (i.e., 3 or 6 wt.% of NaMt or CuMt or TiMt) the higher the obtained strength (σ_uts_ (MPa)). The increase of strength with nanoclay addition is typical for polymer/biopolymer clay reinforced films [27,49,50,51]. The highest strength is observed for CuMt-based nanocomposite films which show a strong intercalated nanostructure according to XRD results. The lowest strength is observed for TiMt-based nanocomposite films which show the “weaker” intercalated nanocomposite structure according to XRD results. According to literature reports [52], tensile strength and elastic modulus of some common plastic films used in food packaging are in the range 6–177 MPa and 150–2900 MPa respectively. The same source reports the tensile strength of PVOH in the range of 39–118 MPa and elastic modulus at 2900 MPa. It is obvious that the values of materials in our study are in good agreement (and in some cases better) with those in the literature.

### 3.4. Water Sorption

From the water sorption data in Table 2, it is obvious that for all tested films low water sorption values are observed. PVOH and nanoclay addition slightly increase the % water sorption. Low water sorption values are attributed to the hot pressing technique followed in the formation process of the films [30]. PVOH and nanoclay addition lead to an increase of water sorption values which could be attributed to the excess amount of hydroxyl groups (OH) in the PVOH/CS blends [27,53] as well as to the uncovered O or OH adsorption sites in clay-based nanocomposite films. These sites exhibit a strong trend to interact with water [54].

### 3.5. Barrier Properties

Calculated WVTR values as well as obtained OTR values for all tested films are listed in Table 2. The same trends are observed for both WVTR and OTR values. PVOH causes a significant decrease of both WVTR and OTR values following previous reports. As reported to the literature, PVOH exhibits a good compatibility with CS and the addition of PVOH act as plasticizer which improves water and oxygen barrier [27,47,48]. Further decrease of both WVTR and OTR values is observed with all nanoclays addition. It is well known that nanoclay addition increases gas barrier properties by increasing the tortuosity of the path that one gas molecule must go through to permeate the polymer or biopolymer matrix [29,50,55,56]. XRD results, water diffusion coefficient measurements D_WV_, and oxygen permeability coefficient measurements Pe_O2_ indicate that the highest increase in water/oxygen barrier is observed for CuMt-based nanocomposite films and the lowest increase in water/oxygen barrier is observed for TiMt-based nanocomposite films. According to literature reports [52], Water-Vapor Permeability and Oxygen Permeability of some common plastic films used in food packaging are in the range 0.001 to 0.3 g∙m^−2^∙day∙Pa^−1^ and 10^−4^ to 44.76 × 10^−4^ cc∙m^−2^∙day∙Pa^−1^ respectively. The same source reports the Water-Vapor Permeability and Oxygen Permeability of PVOH at 0.3 g∙m^−2^∙day∙Pa^−1^ and 3 × 10^−4^ cc∙m^−2^∙day∙Pa^−1^, respectively. It is obvious that the values of materials in our study are superior compared to those in the literature.

### 3.6. Antimicrobial Activity

The antimicrobial effect of nanoreinforcement chitosan (CS)/poly-vinyl-alcohol (PVOH)-based packaging films is shown in Table 3. The antibacterial activity of films was tested against four food pathogenic bacteria i.e., *Escherichia coli, Staphylococcus aureus, Salmonella enterica*, and *Listeria monocytogenes*. The inhibitory activity was measured based on the diameter of the clear inhibition zone. If no surrounding clear zone was observed, then it could be assumed that there is no inhibitory zone, and the diameter was valued as zero. Moreover, the area of the films which was in direct contact with the studied bacteria was used to evaluate bacteria growth inhibition under the film discs. Such bacteria were inoculated on the agar surface. The results of the studied CS/PVOH/CuMt and CS/PVOH/TiMt chitosan films were compared with the results of the CS, CS/PVOH, and CS20PVOH/NaMt films.

Considering the surrounding clear zone, the CS and CSPVOH/6NaMt chitosan films did not exhibit an inhibitory effect against all tested microorganisms. However, no bacterial growth of *E. coli, S. aureus* and, *S. enterica* was observed under the films. At the same time, there was no antibacterial effect in the contact area for *L. monocytogenes*. The CS/PVOH film exhibited antibacterial activity and formed an inhibitory zone of 3.95 mm for *E. coli*, 4.35 mm for *S. aureus*, and 3.00 mm for *S. enterica*. On contrary, there was no effect of CS/PVOH films *on L. monocytogenes*, neither in its inhibitory zone nor under the film.

Results indicated that the antibacterial activity in most cases was generated by the presence of CuMt and TiMt nanostructures. The formation zone which was clearly observed around the disc containing nanostructures indicated their antibacterial nature. It seems that the inhibitory zone was enhanced by the increase of nanostructures amount which was incorporated into chitosan films; however, this increment did not significantly improve the antimicrobial effect. Specifically, the CS/PVOH/6CuMt and CS/PVOH/6TiMt films exhibited higher antibacterial activity against all tested bacteria compared to the antibacterial activity of the CS/PVOH/3CuMt and CS/PVOH/3TiMt films respectively but this difference was not statistically significant (*p* < 0.05).

The inhibitory zones for CS/PVOH/6CuMt film against *E. coli* (5.50 mm), *S. aureus* (5.00 mm), and *L. monocytogenes* (5.10 mm) were markedly higher, followed by the inhibitory zones of CS/PVOH/6TiMt film. Copper ions interact with the negatively charged surface of the bacterial cell, leading to protein denaturation and cell death and also affecting some bacterial biochemical processes [57].

In all cases, the nano-enforcement films showed better antimicrobial activity compared to the CS/PVOH films. An exception was the CS/PVOH/3TiMt film which exhibited lower antibacterial activity against *S. aureus*. Once again, the differences were not statistically significant.

It is known that chitosan possesses some antimicrobial activities coming from the interaction of the positively charged ammonium (NH_4_^+^) of the amino glucose units with the negatively charged compounds in the bacteria cell wall. The result of this interaction is the disruption of the cell functioning and the breakdown of the bacterial outer membrane which causes leakage of vital intracellular constituents and leads to bacterial death [58]. The studied chitosan films (CS) which were used as control material as well as the CSPVOH/6NaMt films, did not exhibit any migrated antimicrobial activity against the tested bacteria. Possibly, the use of the NaMt leads to a reduced diffusion of the PVOH into the agar and consequently to the lack of surrounding inhibiting zones. Thus, it could be concluded that the general effectiveness of the nano-enforcement chitosan films is due to the combined efficiency of the PVOH and CuMt/TiMt components which are added to the non-migrated antimicrobial potency of chitosan. Moreover, the different interactions between the composites of the films may result in varying degrees of inhibition.

In other publications, it was reported that antimicrobial activity is dependent on many parameters such as the size, shape, polarity, chemical structure and the crosslinking level of the material, etc. [59]. It is also crucial to mention that the antimicrobial activity of a nanostructure is strongly correlated with the bacterial strain, nanoparticle type/size, type of growth media, and the initial bacterial cell concentration [15].

### 3.7. Statistical Analysis of the Experimental Data

As it obvious from the literature it is usual in such studies to measure every property at least three times under the same conditions to achieve accurate mean values standard deviation values and standard error [60,61,62]. Moreover, it is usual to test the inequality of mean values between all kind of samples to promote the better material which exhibits superior properties. This test is carried out using the statistical hypothesis H0: (Mean values could be assumed as equal) and is performed for supporting the hypothesis that considering different samples, every property has a statistically different mean value. All the experimental data for E, σ_uts_, ε_b_%, WVTR, % water sorption, OTR, *E. coli, S. aureus, S. enterica,* and *L. monocytogenes* were statistically treated using the statistical software SPSS ver. 20. Statistical tests were carried out assuming the common value C.I. = 95% for the confidential intervals and thus a significance level of a = 0.05. Results for mean values and standard deviation of the above-mentioned parameters are presented in Table 2 and Table 3.

Because the normality tests indicated that some datasets cannot be assumed as normal distributions, the t-test for independent samples method could not be used and the nonparametric Mann–Whitney U test for independent samples was used instead to test the H0 hypothesis. According to clarifications reported elsewhere [37], for *Sig.* < a (i.e., H0 is false), Equation (8) was developed for the calculation of an empirical factor we call “inequality assurance” (0 < IA < 1). The smaller the (*Sig*.) value, compared to significance level value (*a*), the more assured that the mean values are statistically unequal.

(8) $$IA=\frac{a-Sig.}{a}*100$$

For *Sig.* > *a* (i.e., H0 is true), Equation (9) was developed for the calculation of an empirical factor we call “equality assurance” (0 < *EA* < 1). The bigger the (*Sig*.) value, compared to significance level value (*a*), the more assured that the mean values are statistically equal.

(9) $$EA=\frac{Sig.-a}{1-a}*100$$

According to Equation (8) in all cases where the hypothesis H0 was false the “inequality assurance” of the mean values was *IA* > 88%, while according to Equation (9) in all cases where the hypothesis H0 was true the “equality assurance” of the mean values was *EA* > 82%. Table 4 presents the results of such statistical analysis i.e., for each parameter, the overall numerical mean value of each samples group with statistically equal mean values of this parameter.

## 4. Conclusions

As an overall conclusion, we could say that CS/PVOH/CuMt, and CS/PVOH/TiMt nanocomposites are promising materials for active food packaging films. In addition, this is because they were developed using totally biodegradable precursors (i.e., PVOH, chitosan, clay) some of which are bio-byproducts (i.e., chitosan) and some others are natural raw materials (i.e., clays). Between these two nanocomposite films, CS/PVOH/CuMt is the most promising. Both materials exhibit improved or similar oxygen permeability, water-vapor diffusivity, and mechanical properties compared to the relevant values of commercial packaging films. These good results are achieved via the good mixing of components which is confirmed by the FTIR measurements and via the good intercalation of clays which is confirmed by the XRD measurements. The environmental fingerprint of the films which were developed in this study was positive because we achieved antimicrobial and antioxidant properties avoiding the use of chemicals to obtain extended shelf-life for foods and improved food preservation. CS/PVOH/NaMt, CS/PVOH/CuMt, and CS/PVOH/TiMt films exhibited strong antimicrobial activity in the direct contact area with the agar but not in the surrounding area. This means that the use of NaMt or CuMt or TiMt clay reduces drastically the migration of the PVOH or the CS and the diffusion of the CS and PVOH into the agar.

## Figures and Tables

**Table 1:** Symbol of samples and amounts of CS, PVOH, CuMt, and TiMt used for the preparation of all CS/PVOH/CuMt and CS/PVOH/TiMt active films.

Symbol of Samples	CS gr-wt.%	PVOH gr-wt.%	NaMt gr-wt.%	CuMt gr-wt.%	TiMt gr-wt.%
CS	2.0-100	-		-	-
CS/PVOH	2.0-100	0.25-20		-	-
CS/PVOH			0.08-3		
CS/PVOH/3NaMt			0.17-6		
CS/PVOH/6NaMt					
CS/PVOH/3CuMt	2.0-100	0.5-20		0.08-3	-
CS/PVOH/6CuMt	2.0-100	0.59		0.17-6	-
CS/PVOH/3TiMt	2.0-100	0.5-20		-	0.08-3
CS/PVOH/6TiMt	2.0-100	0.59-20		-	0.17-6

CS is chitosan; PVOH is Poly-vinyl-alcochol; CuMt is copper-montmorillonite composite; TiMt is titanium-montmorillonite composite; CS/PVOH/CuMt is is the final film containing chitosan/copper-montmorillonite nanostructure in poly-vinyl-alcochol matrix; CS/PVOH/TiMt is is the final film containing chitosan/titanium-montmorillonite nanostructure in poly-vinyl-alcochol matrix.

**Table 2:** Calculated values of Young’s (E) Modulus, ultimate tensile strength (σ_uts_) and % strain at break (ε_b_).

Symbol of Samples	E Modulus (MPa)	σ_uts_ (MPa)	ε_b_%	Water Sorption	WVTR (g∙m^−2^∙day^−1^)	D_WV_ (cm^2^∙s^−1^)	OTR (cm^3^∙m^−2^∙day^−1^)	Pe_O2_ (cm^2^∙s^−1^)
CS	3274 ± 62	92.2 ± 3.5	10.8 ± 1.4	157 ± 5	15.5 ± 0.2	7.06 × 10^−12^	161.0 ± 2.4	6.52 × 10^−10^
CS/PVOH	2920 ± 105	86.4 ± 3.4	14.5 ± 0.8	162 ± 7	6.3 ± 0.2	2.87 × 10^−12^	70.0 ± 1.9	2.84 × 10^−10^
CS/PVOH/3NaMt	3200 ± 83	91.8 ± 4.2	7.9 ± 1.7	172 ± 4	6.0 ± 0.2	2.73 × 10^−12^	59.6 ± 1.4	2.41 × 10^−10^
CS/PVOH/6NaMt	3340 ± 51	96.4 ± 2.8	6.4 ± 1.2	176 ± 6	5.6 ± 0.1	2.55 × 10^−12^	52.3 ± 1.2	2.12 × 10^−10^
CS/PVOH/3CuMt	3270 ± 89	93.8 ± 4.2	7.4 ± 1.8	171 ± 5	5.7 ± 0.2	2.60 × 10^−12^	58.6 ± 1.3	2.37 × 10^−10^
CS/PVOH/6CuMt	3380 ± 51	97.1 ± 2.8	6.1 ± 1.2	181 ± 7	5.5 ± 0.1	2.51 × 10^−12^	50.6 ± 1.1	2.05 × 10^−10^
CS/PVOH/3TiMt	2930 ± 89	87.1 ± 4.2	7.1 ± 1.8	180 ± 6	6.1 ± 0.2	2.80 × 10^−12^	66.8 ± 1.4	2.71 × 10^−10^
CS/PVOH/6TiMt	3080 ± 51	89.1 ± 2.8	6.1 ± 1.2	186 ± 7	5.9 ± 0.1	2.69 × 10^−12^	57.2 ± 1.2	2.32 × 10^−10^

WVTR is water-vapor transmission rate; DWV is water diffusion coefficient; OTR is oxygen transmission rate; Pe_O2_ is oxygen permeability coefficient values.

**Table 3:** Antimicrobial activity of active films against food pathogenic bacteria *E. coli*, *S. aureus*, *S. enterica* and *L. monocytogenes*.

Film Material	*E. coli*		*S. aureus*		*S. enterica*		*L. monocytogenes*	
	Inhibition ^a^	Contact Area ^b^	Inhibition ^a^	Contact Area ^b^	Inhibition ^a^	Contact Area ^b^	Inhibition ^a^	Contact Area ^b^
CS	0.00	-	0.00	-	0.00	-	0.00	+
CS/PVOH	3.95 ± 0.07	-	4.35 ± 0.49	-	3.00 ± 0.00	-	0.00	+
CS/PVOH/6NaMt	0.00	-	0.00	-	0.00	-	0.00	+
CS/PVOH/3CuMt	4.75 ± 0.35	-	4.95 ± 0.07	-	3.50 ± 0.71	-	3.00 ± 1.41	-
CS/PVOH/6CuMt	5.50 ± 0.71	-	5.00 ± 0.14	-	3.50 ± 0.71	-	5.10 ± 0.14	-
CS/PVOH/3TiMt	3.50 ± 0.71	-	2.50 ± 0.71	-	3.15 ± 0.07	-	3.00 ± 1.41	-
CS/PVOH/6TiMt	4.05 ± 0.07	-	4.45 ± 0.64	-	3.50 ± 0.71	-	4.50 ± 0.42	-

^a^ Diameter of clear zone. Inhibitory zone surrounding film discs measured in mm after the subtraction of the disc diameter (6 mm). ^b^ Contact area of film discs with the agar surface (under the film disc). (+) indicates bacterial growth in the area, (-) indicates no bacterial growth in the area. Results expressed as mean ± standard deviation (*n* = 3).

**Table 4:** Statistical mean values of parameters E. Modul., σ_uts_, ε_b_%, Wat. Sorpt., WVTR, OTR, *E. coli, S. aureus, S. enterica,* and *L. monocytogenes* for all kind of developed films.

	CS	CS/PVOH	CS/PVOH 3NaMt	CS/PVOH 6NaMt	CS/PVOH 3CuMt	CS/PVOH 6CuMt	CS/PVOH 3TiMt	CS/PVOH 6TiMt
E Modulus	3248	2925	3248	3360	3248	3360	2925	3080
σ_uts_	92.6	86.8	92.6	96.8	92.6	96.8	86.8	89.1
ε_b_%	10.8	14.5	7.5	6.2	7.5	6.2	7.5	6.2
Water Sorpt.	157	162	173	173	173	181	181	186
WVTR	15.5	6.3	6.0	5.6	5.6	5.6	6.0	6.0
OTR	161.0	70.0	58.5	51.5	58.5	51.5	66.8	58.5
*E. coli*	0.00	4.00	0.00	0.00	4.75	5.50	3.50	4.00
*S. aureus*	0.00	4.40	0.00	0.00	4.98	4.98	2.50	4.40
*S. enterica*	0.00	3.00	0.00	0.00	3.50	3.50	3.15	3.50
*L. monocytogenes*	0.00	0.00	0.00	0.00	3.00	5.10	3.00	4.50

## Data Availability

The datasets generated for this study are available on request to the corresponding author.

## References

[1] Dainelli D., Gontard N., Spyropoulos D., Zondervan-van den Beuken E., Tobback P. (2008). Active and intelligent food packaging: Legal aspects and safety concerns. Trends Food Sci. Technol..

[2] Soltani Firouz M., Mohi-Alden K., Omid M. (2021). A critical review on intelligent and active packaging in the food industry: Research and development. Food Res. Int..

[3] de Carvalho A.P.A., Conte Junior C.A. (2020). Green strategies for active food packagings: A systematic review on active properties of graphene-based nanomaterials and biodegradable polymers. Trends Food Sci. Technol..

[4] Haghighi H., Licciardello F., Fava P., Siesler H.W., Pulvirenti A. (2020). Recent advances on chitosan-based films for sustainable food packaging applications. Food Packag. Shelf Life.

[5] Priyadarshi R., Rhim J.-W. (2020). Chitosan-based biodegradable functional films for food packaging applications. Innov. Food Sci. Emerg. Technol..

[6] Chawla R., Sivakumar S., Kaur H. (2021). Antimicrobial edible films in food packaging: Current scenario and recent nanotechnological advancements- a review. Carbohydr. Polym. Technol. Appl..

[7] Azlin-Hasim S., Cruz-Romero M.C., Cummins E., Kerry J.P., Morris M.A. (2016). The potential use of a layer-by-layer strategy to develop LDPE antimicrobial films coated with silver nanoparticles for packaging applications. J. Colloid Interface Sci..

[8] Shankar S., Khodaei D., Lacroix M. (2021). Effect of chitosan/essential oils/silver nanoparticles composite films packaging and gamma irradiation on shelf life of strawberries. Food Hydrocoll..

[9] Wang W., Yu Z., Alsammarraie F.K., Kong F., Lin M., Mustapha A. (2020). Properties and antimicrobial activity of polyvinyl alcohol-modified bacterial nanocellulose packaging films incorporated with silver nanoparticles. Food Hydrocoll..

[10] Yin M., Lin X., Ren T., Li Z., Ren X., Huang T.-S. (2018). Cytocompatible quaternized carboxymethyl chitosan/poly(vinyl alcohol) blend film loaded copper for antibacterial application. Int. J. Biol. Macromol..

[11] Mousazadeh S., Ehsani A., Moghaddas Kia E., Ghasempour Z. (2021). Zinc oxide nanoparticles and periodate oxidation in developing pH-sensitive packaging film based on modified gelatin. Food Packag. Shelf Life.

[12] Kaewklin P., Siripatrawan U., Suwanagul A., Lee Y.S. (2018). Active packaging from chitosan-titanium dioxide nanocomposite film for prolonging storage life of tomato fruit. Int. J. Biol. Macromol..

[13] Siripatrawan U., Kaewklin P. (2018). Fabrication and characterization of chitosan-titanium dioxide nanocomposite film as ethylene scavenging and antimicrobial active food packaging. Food Hydrocoll..

[14] Busolo M.A., Lagaron J.M. (2012). Oxygen scavenging polyolefin nanocomposite films containing an iron modified kaolinite of interest in active food packaging applications. Innov. Food Sci. Emerg. Technol..

[15] Bagchi B., Kar S., Dey S.K., Bhandary S., Roy D., Mukhopadhyay T.K., Das S., Nandy P. (2013). In situ synthesis and antibacterial activity of copper nanoparticle loaded natural montmorillonite clay based on contact inhibition and ion release. Colloids Surf B Biointerfaces.

[16] Hu C.H., Xu Z.R., Xia M.S. (2005). Antibacterial effect of Cu^2+^-exchanged montmorillonite on aeromonas hydrophila and discussion on its mechanism. Vet. Microbiol..

[17] He H.P., Guo J.G., Xie X.D., Peng J.L. (2001). Location and migration of cations in Cu^2+^-adsorbed montmorillonite. Environ. Int..

[18] Malachová K., Praus P., Rybková Z., Kozák O. (2011). Antibacterial and antifungal activities of silver, copper and zinc montmorillonites. Appl. Clay Sci..

[19] Mitsudome T., Matsuno T., Sueoka S., Mizugaki T., Jitsukawa K., Kaneda K. (2012). Titanium cation-exchanged montmorillonite as an active heterogeneous catalyst for the beckmann rearrangement under mild reaction conditions. Tetrahedron Lett..

[20] Kawabata T., Kato M., Mizugaki T., Ebitani K., Kaneda K. (2003). Highly efficient deprotection of acetals by titanium cation-exchanged montmorillonite as a strong solid acid catalyst. Chem. Lett..

[21] Sterte J. (1986). Synthesis and properties of titanium oxide cross-linked montmorillonite. Clays Clay Miner..

[22] Bruna J.E., Galotto M.J., Guarda A., Rodríguez F. (2014). A novel polymer based on MtCu^2+^/cellulose acetate with antimicrobial activity. Carbohydr Polym.

[23] Martucci J.F., Ruseckaite R.A. (2017). Antibacterial activity of gelatin/copper (II)-exchanged montmorillonite films. Food Hydrocoll..

[24] Bruna J.E., Peñaloza A., Guarda A., Rodríguez F., Galotto M.J. (2012). Development of MtCu^2+^/LDPE nanocomposites with antimicrobial activity for potential use in food packaging. Appl. Clay Sci..

[25] Effects of Exchange Titanium Cations on the Pore Structure and Adsorption Characteristics of Montmorillonite | Semantic Scholar. https://www.semanticscholar.org/paper/effects-of-exchange-titanium-cations-on-the-pore-of-Huang-Lee/7abbbfcd11812309ee9bc2442b6adcaf1321f6d9.

[26] Giannakas A., Grigoriadi K., Leontiou A., Barkoula N.-M., Ladavos A. (2014). Preparation, characterization, mechanical and barrier properties investigation of chitosan–clay nanocomposites. Carbohydr. Polym..

[27] Giannakas A., Vlacha M., Salmas C., Leontiou A., Katapodis P., Stamatis H., Barkoula N.-M., Ladavos A. (2016). Preparation, characterization, mechanical, barrier and antimicrobial properties of chitosan/PVOH/clay nanocomposites. Carbohydr. Polym..

[28] Giannakas A., Patsaoura A., Barkoula N.-M., Ladavos A. (2017). A novel solution blending method for using olive oil and corn oil as plasticizers in chitosan based organoclay nanocomposites. Carbohydr. Polym..

[29] Vlacha M., Giannakas A., Katapodis P., Stamatis H., Ladavos A., Barkoula N.-M. (2016). On the efficiency of oleic acid as plasticizer of chitosan/clay nanocomposites and its role on thermo-mechanical, barrier and antimicrobial properties—Comparison with glycerol. Food Hydrocoll..

[30] Grigoriadi K., Giannakas A., Ladavos A.K., Barkoula N.-M. (2015). Interplay between processing and performance in chitosan-based clay nanocomposite films. Polym. Bull..

[31] Giannakas A.E., Salmas C.E., Karydis-Messinis A., Moschovas D., Kollia E., Tsigkou V., Proestos C., Avgeropoulos A., Zafeiropoulos N.E. (2021). Nanoclay and polystyrene type efficiency on the development of polystyrene/montmorillonite/oregano oil antioxidant active packaging nanocomposite films. Appl. Sci..

[32] Giannakas A.E., Salmas C.E., Leontiou A., Baikousi M., Moschovas D., Asimakopoulos G., Zafeiropoulos N.E., Avgeropoulos A. (2021). Synthesis of a novel chitosan/basil oil blend and development of novel low density poly ethylene/chitosan/basil oil active packaging films following a melt-extrusion process for enhancing chicken breast fillets shelf-life. Molecules.

[33] Salmas C.E., Giannakas A.E., Baikousi M., Leontiou A., Siasou Z., Karakassides M.A. (2021). Development of poly(L-lactic acid)/chitosan/basil oil active packaging films via a melt-extrusion process using novel chitosan/basil oil blends. Processes.

[34] Salmas C., Giannakas A., Katapodis P., Leontiou A., Moschovas D., Karydis-Messinis A. (2020). Development of ZnO/Na-montmorillonite hybrid nanostructures used for PVOH/ZnO/Na-montmorillonite active packaging films preparation via a melt-extrusion process. Nanomaterials.

[35] Giannakas A., Giannakas A., Ladavos A. (2012). Preparation and characterization of polystyrene/organolaponite nanocomposites. Polym. Plast. Technol. Eng..

[36] Giannakas A., Salmas C., Leontiou A., Tsimogiannis D., Oreopoulou A., Braouhli J. (2019). Novel LDPE/chitosan rosemary and melissa extract nanostructured active packaging films. Nanomaterials.

[37] Giannakas A., Stathopoulou P., Tsiamis G., Salmas C. (2019). The effect of different preparation methods on the development of chitosan/thyme oil/montmorillonite nanocomposite active packaging films. J. Food Process. Preserv..

[38] Edible Food Packaging: Materials and Processing Technologies—1st Edition. https://www.routledge.com/edible-food-packaging-materials-and-processing-technologies/cerqueira-pereira-ramos-teixeira-vicente/p/book/9781482234169.

[39] Units of Gas Permeability Constants—Yasuda—1975—Journal of Applied Polymer Science—Wiley Online Library. https://onlinelibrary.wiley.com/doi/abs/10.1002/app.1975.070190915.

[40] Balouiri M., Sadiki M., Ibnsouda S.K. (2016). Methods for in vitro evaluating antimicrobial activity: A review. J. Pharm. Anal..

[41] Mekhzoum M.E.M., Benzeid H., Qaiss A.E.K., Essassi E.M., Bouhfid R. (2016). Copper(I) confined in interlayer space of montmorillonite: A highly efficient and recyclable catalyst for click reaction. Catal. Lett..

[42] Gartner C., López B.L., Sierra L., Graf R., Spiess H.W., Gaborieau M. (2011). Interplay between structure and dynamics in chitosan films investigated with solid-state NMR, dynamic mechanical analysis, and X-ray diffraction. Biomacromolecules.

[43] Naveen Kumar H.M.P., Prabhakar M.N., Venkata Prasad C., Madhusudhan Rao K., Ashok Kumar Reddy T.V., Chowdoji Rao K., Subha M.C.S. (2010). Compatibility studies of chitosan/PVA blend in 2% aqueous acetic acid solution at 30 °C. Carbohydr. Polym..

[44] Zhang L., Wang H., Jin C., Zhang R., Li L., Li X., Jiang S. (2017). Sodium lactate loaded chitosan-polyvinyl alcohol/montmorillonite composite film towards active food packaging. Innov. Food Sci. Emerg. Technol..

[45] Ming-li C., Ying-bo Z., Yong-fu Y. (2002). Preparation and microstructure of Al-pillared interlayered montmorillonite. J. Wuhan Univ. Technol.-Mat. Sci. Edit..

[46] Zhang Q., Zhang Y., Liu S., Wu Y., Zhou Q., Zhang Y., Zheng X., Han Y., Xie C., Liu N. (2021). Adsorption of deoxynivalenol by pillared montmorillonite. Food Chem..

[47] Srinivasa P.C., Ramesh M.N., Kumar K.R., Tharanathan R.N. (2003). Properties and sorption studies of chitosan-polyvinyl alcohol blend films. Carbohydr. Polym..

[48] Costa-Júnior E.S., Barbosa-Stancioli E.F., Mansur A.A.P., Vasconcelos W.L., Mansur H.S. (2009). Preparation and characterization of chitosan/poly(vinyl alcohol) chemically crosslinked blends for biomedical applications. Carbohydr. Polym..

[49] Giannakas A.E., Leontiou A.A. (2018). Montmorillonite composite materials and food packaging. Composites Materials for Food Packaging.

[50] Arunvisut S., Phummanee S., Somwangthanaroj A. (2007). Effect of clay on mechanical and gas barrier properties of blown film LDPE/clay nanocomposites. J. Appl. Polym. Sci..

[51] Vicente A.A. (2009). Food hydrocolloids chitosan/clay films ‘Properties as affected by biopolymer and clay micro/nanoparticles’ concentrations. Food Hydrocoll..

[52] Bastarrachea L., Dhawan S., Sablani S.S. (2011). Engineering properties of polymeric-based antimicrobial films for food packaging: A review. Food Eng. Rev..

[53] Hyder M.N., Chen P. (2009). Pervaporation dehydration of ethylene glycol with chitosan-poly(vinyl alcohol) blend membranes: Effect of CS-PVA blending ratios. J. Membr. Sci..

[54] Lavorgna M., Piscitelli F., Mangiacapra P., Buonocore G.G. (2010). Study of the combined effect of both clay and glycerol plasticizer on the properties of chitosan films. Carbohydr. Polym..

[55] Choudalakis G., Gotsis A.D. (2009). Permeability of polymer/clay nanocomposites: A review. Eur. Polym. J..

[56] Hosseinkhanli H., Sharif A., Aalaie J., Khalkhali T., Akhlaghi S. (2013). Oxygen permeability and the mechanical and thermal properties of (low-density polyethylene)/poly (ethylene-co-vinyl acetate)/organoclay blown film nanocomposites. J. Vinyl Addit. Technol..

[57] Azam A., Ahmed A.S., Oves M., Khan M., Memic A. (2012). Size-dependent antimicrobial properties of CuO nanoparticles against gram-positive and -negative bacterial strains. Int. J. Nanomed..

[58] Shahidi F., Arachchi J.K.V., Jeon Y.-J. (1999). Food applications of chitin and chitosans. Trends Food Sci. Technol..

[59] Cagri A., Ustunol Z., Ryser E.T. (2001). Antimicrobial, mechanical, and moisture barrier properties of low pH whey protein-based edible films containing p-aminobenzoic or sorbic acids. J. Food Sci..

[60] García A.V., Álvarez-Pérez O.B., Rojas R., Aguilar C.N., Garrigós M.C. (2020). Impact of olive extract addition on corn starch-based active edible films properties for food packaging applications. Foods.

[61] Amor G., Sabbah M., Caputo L., Idbella M., De Feo V., Porta R., Fechtali T., Mauriello G. (2021). Basil essential oil: Composition, antimicrobial properties, and microencapsulation to produce active chitosan films for food packaging. Foods.

[62] Kumari N., Bangar S.P., Petrů M., Ilyas R.A., Singh A., Kumar P. (2021). Development and characterization of fenugreek protein-based edible film. Foods.

